# A real-world economic analysis of biologic therapies for psoriatic arthritis in Italy: results of the CHRONOS observational longitudinal study

**DOI:** 10.1186/s12913-022-08954-8

**Published:** 2022-12-16

**Authors:** Emanuela Zagni, Micol Frassi, Giuseppa Pagano Mariano, Enrico Fusaro, Claudia Lomater, Patrizia Del Medico, Florenzo Iannone, Rosario Foti, Massimiliano Limonta, Antonio Marchesoni, Bernd Raffeiner, Ombretta Viapiana, Walter Grassi, Rosa Daniela Grembiale, Giuliana Guggino, Antonino Mazzone, Enrico Tirri, Roberto Perricone, Pier Carlo Sarzi Puttini, Salvatore De Vita, Fabrizio Conti, Alessandro Zullo, Lucia Simoni, Martina Fiocchi, Roberto Orsenigo, Delia Colombo

**Affiliations:** 1 Value &Access, Novartis Farma S.p.A, Largo Umberto Boccioni, 1 21040 Varese, Origgio Italy; 2 Rheumatology and Clinical Immunology Unit, ASST Spedali Civili, Brescia, Italy; 3 UOSD Reumatologia GOM “Bianchi-Melacrino-Morelli”, Reggio Calabria, Italy; 4 Rheumatology Dept. AOU Città della Salute e della Scienza di Torino, Turin, Italy; 5 SSDDU Reumatologia ASO Ordine Mauriziano, Turin, Italy; 6 Ospedale civile, Civitanova Marche, Italy; 7 U.O. Reumatologia, A.O.U. Policlinico Consorziale, Bari, Italy; 8 UOS Reumatologia, A.O.U. Policlinico -Vittorio Emanuele, Catania, Italy; 9 UOSD Reumatologia, ASST Papa Giovanni XXIII, Bergamo, Italy; 10 Department of Rheumatology, ASST Gaetano Pini-CTO, Milan, Italy; 11 Ospedale Centrale di Bolzano, Bolzano, Italy; 12 U.O.C. Reumatologia, AOUI Verona Borgo Roma, Verona, Italy; 13 Policlinico A. Murri, Jesi, Italy; 14 U.O. Medicina Interna, A.O.U. Mater Domini, Catanzaro, Italy; 15 U.O. Reumatologia, A.O.U. Policlinico Giaccone, Palermo, Italy; 16 Medicina Interna MAC area medica, Ospedale Civile di Legnano, Legnano, Italy; 17 Ospedale San Giovanni Bosco, Naples, Italy; 18 U.O.C. Reumatologia, Policlinico Tor Vergata, Rome, Italy; 19 U.O.C. Reumatologia, ASST FBF Sacco, Milan, Italy; 20 Clinica Reumatologica, ASUIUD, Udine, Italy; 21 U.O.C. Reumatologia, Azienda Policlinico Umberto I, Rome, Italy; 22 MediNeos Observational Research, Modena, Italy

**Keywords:** Psoriatic arthritis, Biologics, Tumor necrosis factor, Secukinumab, Costs, Cost-per-responder, Real-world

## Abstract

**Background:**

Psoriatic arthritis (PsA) is a chronic, immune-mediated, spondyloarthropathy characterised by musculoskeletal signs and symptoms with associated joint pain and tenderness. The average worldwide PsA prevalence is 133/100,000, while in the Italian population is 90–420/100,000.

Traditionally, nonsteroidal anti-inflammatory drugs, glucocorticoid, and disease-modifying antirheumatic drugs have been used in the treatment of PsA. However, for those patients who are not adequately controlled with conventional therapies, the new biologics compounds represent a valid option. Biologic therapies have been shown to be more effective but also more expensive than conventional systemic treatments. Based on the CHRONOS study, the economic analyses presented in this paper aim to assess the annualised direct costs and the cost-per-responder of biologics in a real-world context assuming the Italian National Health System perspective.

**Methods:**

The economic assessments were carried out on the overall cohort of patients, and on the tumour necrosis factor alpha inhibitors (TNFi) and the secukinumab subgroup, the most prescribed biologic therapies within the CHRONOS study.

**Results:**

The annual economic impact of PsA in the overall group was €12,622, €11,725 in the secukinumab subgroup, and €12,791 in the TNFi subgroup. Biologics absorbed the main expenditure costs in the treatment of PsA accounting for about the 93% of total costs. At 6 months, secukinumab performed better in all the considered outcomes: cost-per-responder according to EULAR DAS28 and ACR50 response criteria were €12,661- €28,975, respectively, while they were €13,356 - €33,368 in the overall cohort and €13,138 - €35,166 in the TNFi subgroup. At 12 months secukinumab remained the subgroup with the lowest cost-per-responder ratio in EULAR DAS28 and ACR50 response criteria, while TNFi subgroup was the lowest one considered the ACR20.

**Conclusion:**

Despite some potential methodological limitations, our cost-per-response analysis provides physicians and payers additional insights which can complement the traditional risk-benefit profile assessment and drive treatment decisions.

**Supplementary Information:**

The online version contains supplementary material available at 10.1186/s12913-022-08954-8.

## Background

Psoriatic arthritis (PsA) is a seronegative spondyloarthropathy characterised by musculoskeletal signs (arthritis, enthesitis, spondylitis) and symptoms (fatigue and stiffness) with associated joint pain and tenderness [1, 2].

The worldwide PsA prevalence and incidence rates in the general population are 133 every 100,000 subjects (95% CI, 107–164 every 100,000 subjects) and 83 every 100,000 subjects per year (95% CI, 41–167 every 100,000 subjects per year), respectively [3]. As regards Italy, a recent study estimated an overall PsA prevalence of 90–420 cases every 100,000 subjects [4].

As for other chronic, systemic inflammatory disorders, management of PsA aims to suppress inflammation to minimise articular and dermatologic symptoms, prevent structural damage, and improve quality of life of affected patients [5].

A wide spectrum of pharmacologic treatments is available for PsA. Mild forms of arthritis are generally treated with nonsteroidal anti-inflammatory drugs (NSAIDs) and intraarticular glucocorticoid injections. Patients with moderate-severe PsA are treated with immunomodulatory therapies, which include oral small molecules such as methotrexate, leflunomide, sulfasalazine, cyclosporine (and more recently, apremilast), defined as disease-modifying antirheumatic drugs (DMARDs). For PsA patients who are not adequately controlled with conventional DMARDs therapy, the new biologic therapies are a valid option. TNFi (infliximab, adalimumab, etanercept, golimumab, certolizumab) are the first-line biologics in the treatment of PsA patients [6, 7]. Other biologic options include the interleukin-12/23 inhibitors (IL12/23: ustekinumab), and interleukin 17 inhibitors (IL17: secukinumab, ixekizumab) [7]. Given the complexity in treating PsA patients, several international and national guidelines are currently available to support rheumatologists in daily clinical practice management of PsA [6–8].

Biologic therapies have been shown to be more effective but also more expensive than conventional systemic treatments (DMARDs) [9]. Use of biologic therapies for PsA has increased significantly over the last decades, with consequent increase of National Health Service budget allocated on PsA [10].

At the time of launch of a new treatment, the economic evidence is assessed using models, under the assumption that the efficacy observed in clinical trials would be similar in the “real-world”. Also, evidence of long-term effects is rarely available during the early stage of commercialization. Therefore, long-term simulations are used to predict “lifetime” costs and clinical consequences of treatment use. Finally, health technology assessment agencies and pricing and reimbursement institutions are interested in evaluating the economic implications of new technology adoption in their respective countries [11].

The use of observational research for economic purposes can inform on critical aspects of decision-making, such as budget impact/allocation, cost-effectiveness and cost-utility analysis of new technologies [12]. Given the chronic nature of PsA and the considerable amount of direct costs (drugs costs, inpatient costs for acute care, outpatient costs for regular monitoring) and indirect costs (caregivers, loss of working days) spent in the treatment of PsA [13–16], the analysis of real-world data is important to assess the economic burden of the disease [17].

The CHRONOS (EffeCtiveness of biologic treatments for psoriatic artHRitis in Italy: an ObservatioNal lOngitudinal Study of real-life clinical practice) study was designed to provide real-world evidence (RWE) of the effectiveness of biologic treatments for PsA in the Italian real-life clinical practice; main results of the study are presented elsewhere [17].

The study also collected economic endpoints concerning costs and cost-effectiveness of biologics for PsA. The present paper is aimed to describe the cost-effectiveness of biologic agents as used in clinical practice, with real-life conditions. For this purpose, the annualised overall costs of PsA management, the annualised cost of biologic drugs and the cost-per-responder ratios in the Italian National Health System (NHS) perspective were assessed.

## Methods

### Source of clinical data

#### Observational study design

The CHRONOS study was a multicenter, non-interventional retro-prospective cohort study conducted in 20 Italian rheumatology hospitals, which involved both the use of primary data (registered during the visits in the CHRONOS study) and secondary data (e.g. unit costs of drugs, visits, phototherapy, rehabilitation, collected through the desk research). Retrospective data since initiation of the earliest biologic were collected back at enrolment visit from hospital medical charts or other clinical documents, while prospective data were collected at the enrolment and 6-month (±1 month) follow-up visits, which took place as per normal clinical practice.

Patients aged ≥18 years, with diagnosis of PsA according to the treating rheumatologist, who had initiated a biologic treatment between 24 weeks and 24 months before enrolment visit (retrospective period), were consecutively enrolled, provided they had available data for DAS28 in the retrospective period. Enrolment lasted from September 2018 to September 2019 and end date of data collection was in April 2020. Patients who had interrupted treatment before enrolment could also be included. Detailed description of eligibility and evaluability criteria are reported elsewhere [17].

At the enrolment visit, data since initiation of the earliest biologic were retrospectively collected from hospital medical charts or other clinical documents, while the prospective observational period was 6 months (±1 month), so that each patient was planned by design to have a total of observational period of at least 12 months except in case of early withdrawal. Patients who withdrew from the study were included in the analyses if they had available clinical outcomes.

#### Treatments

In our analyses the “reference biological therapy” was defined as the biologic therapy ongoing at enrolment or taken last before the enrolment visit, in case the patient was no longer assuming a biologic at enrolment. All enrolled patients were treated with at least one of the following biologic therapies: secukinumab (Cosentyx), adalimumab (Humira), adalimumab biosimilar (Amgevita, Imraldi), certolizumab (Cimzia), etanercept (Enbrel), etanercept biosimilar (Benepali, Erelzi), golimumab (Simponi), infliximab (Remicade), infliximab biosimilar (Flixabi, Remsima), ustekinumab (Stelara).

Two types of analyses were conducted: i) overall analysis, on the entire cohort of PsA patients treated with biologic therapies; ii) a post-hoc analysis stratified by secukinumab or TNFis subgroups (TNFi comprising adalimumab (originator and biosimilar), certolizumab, etanercept (originator and biosimilar), golimumab, and infliximab (originator and biosimilar)) given that most patients were treated with these two major treatment groups [17]. All other treatments were not analysed as subgroups in the post-hoc analyses, as their sample size was small, with findings being potentially affected by precision and accuracy issues.

#### Response criteria in the economic analyses

Both DAS28 (Disease Activity Score 28 points) provided by the European League Against Rheumatism (EULAR) and American College of Rheumatology (ACR) response criteria were used as response criteria in our economic analyses. There was consensus among clinicians participating at the study that these two tools were the most used and comprehensive instruments to measure disease activity in PsA. EULAR DAS28 and ACR criteria are clinical composite outcomes developed for rheumatoid arthritis (RA) largely used to determine both disease activity and treatment response in PsA patients [5, 18–20]. DAS28 is frequently used also in Italian clinical practice [21, 22]; it takes into account a 28 tender joint count (range 0–28), a 28 swollen joint count (range 0–28), erythrocyte sedimentation rate (ESR) or C-reactive protein (CRP), and patients’ general health (GH) measured by a visual analogue scale [23]. The EULAR response criteria classify patients as good, moderate or non-responders, using the individual amount of change in the DAS28 and the DAS28 value reached (low, moderate, or high) [24, 25]. Patients who achieved a good or moderate response were considered as responders in our cost-effectiveness analysis.

Achievement of sustained response at 12 months (intended as achievement of DAS28 good/moderate response through all the time points considered; 6 and 12 months) was evaluated as well.

The ACR20–50 criteria are standard measures of the effectiveness of various treatments in clinical trials for RA [5, 19, 20, 26, 27]. ACR20 stands for achievement of 20% improvement in *N* = 68 tender or *N* = 66 swollen joint counts, as well as a 20% improvement in at least three of the other five criteria (patient assessment, physician assessment, pain scale, disability/functional questionnaire, acute phase reactant - erythrocyte sedimentation rate or C-reactive protein -). The same logic applies to the interpretation of ACR50 in which the improvement is set at 50%.

The analyses were performed considering only patients with available EULAR DAS28 and ACR responses at start of biologic therapy and after 6 (or 12) months.

### Source of economic data

#### Resource consumption

The following resource consumption data were collected to determine costs of PsA: i) pharmacological biologic treatments received during the study period, settings of administration (inpatient, ambulatory outpatient, home), frequency of treatment, duration of treatment; ii) pharmacological concomitant adjunctive PsA treatments (topical and non-topical therapies, non-biological therapies) iii) biologic treatment-related adverse events (drugs prescribed, follow-up visits, procedures, hospitalisations to manage the AEs); iv) treatment follow-up/monitoring during the study period (general practitioner -GP- and specialist outpatient visits, laboratory examinations, diagnostic examinations); v) rehabilitative care received during the study period (description of the intervention, number of sessions); vi) other interventions during the study period (hospitalisations due to disease worsening or adverse events, emergency department (ED) accesses, phototherapy, rehabilitation).

#### Costs

The economic analyses were conducted adopting the Italian national healthcare service (NHS or SSN in Italian) perspective: therefore, only direct costs sustained by the Italian SSN were considered and collected.

Ex-manufacturer acquisition costs of therapies and tariffs for outpatient and inpatient services were retrieved from national databases [28, 29].

For systemic biologic therapies, the cost was obtained multiplying the unit cost by the total quantity of drug received by the patient during the observation period, taking into consideration the treatment posology.

The costs of topical therapies for PsA, other pharmacological therapies for PsA, and other relevant concomitant medications to manage adverse events related to biologic treatments, were obtained multiplying the unit cost by the total quantity of drug received by the patient during the observation period. The posology was estimated according to the indications of the Summary of Product Characteristics of each drug.

Phototherapy [28] and rehabilitative [29] care costs were obtained multiplying the unit costs by the total number of received sessions.

ED accesses, and inpatient hospitalisations tariffs were based on the DRG codes; GP visits costs for PsA were assigned according to Garattini et al. [30] (Cost inflated from January 2003 to October 2019 [31]). The costs of specialistic outpatient visits for PsA were assigned according to Italian Ministry of Health, outpatient and hospital tariffs, respectively [28, 29].

The test/procedures/instrumental examination costs were assigned according to Italian Ministry of Health, outpatient tariffs [28].

### Economic analysis

The economic assessment consisted of three different analyses: i) PsA management cost analysis; ii) cost-per-response analysis, iii) cost-per-sustained response analysis.

In the PsA management cost analysis, per-patient costs of therapies and healthcare services were summed and then divided by the duration of observation window (in months) from the start of reference biologic therapy.

In the cost-per-response analysis, the cost-per-patient and the cost-per-responder ratio were calculated. The first one was defined as the total cost spent in the overall population or in a subgroup of patients divided by the number of patients belonging, respectively, to the overall sample or to the related subgroup. The cost-per-responder ratio was calculated to estimate cost-effectiveness of biologic therapies [32] and was defined as the amount of investment required to successfully treat one patient, according to DAS28 and ACR response criteria. The cost-per-responder ratio at 6 (tolerance window: 4 to 9 months) and 12 months (tolerance window: 10 to 15 months) was calculated as the sum of individual costs divided by the number of patients achieving clinical response (in terms of DAS28 and ACR20–50) at the respective time-point. Results of the cost-per-response analysis depend on: i) timeframe (6 months, 12 months); ii) type of outcome used to define the “response” (DAS28, ACR20, ACR50); iii) response rate achieved.

A cost-per- DAS28 good/moderate sustained response analysis at 12 months, was conducted to evaluate consistency of findings in the cost-per-response analyses. The sustained cost-per-responder ratio was defined as the amount of investment required to achieve sustained response. The sustained cost-per-responder ratio at 12 months was calculated as the sum of individual costs divided by the number of patients achieving sustained response (as previously defined).

The previously mentioned indicators were calculated both (i) considering the overall cohort of patients and (ii), as post-hoc analysis, by treatment subgroup (secukinumab and TNFi). The choice of subgroups was guided by sample size reasons and was not intended to conduct a formal statistical comparison, which would have been not in the scope of the study and would have not been possible given the purely observational design of the study. However, the secukinumab was the only large-enough to be assessed as a single-agent subgroup, while the other therapies (mainly TNFi, either originators or biosimilars) were quite small in size to be analysed individually.

## Results

### Patients’ enrolment and subgroups

The study was conducted in 20 Italian rheumatology hospitals, which enrolled *N* = 409 patients; of these, *N* = 10 patients did not meet the CHRONOS study inclusion criteria [17]. Therefore, *N* = 399 patients were evaluated in terms of clinical assessment and healthcare resource use. Socio-demographic and main clinical characteristics at enrolment have been reported in previous publications [17] and are also summarized in the additional file [see Additional file 1]. Mean age at enrolment was 52.4 years (SD 11.6), and *N* = 172 patients (43.1%) were male. *N* = 17 patients (4.3% of the eligible) prematurely discontinued the study (11 were lost to follow-up, 2 became pregnant, 2 due to Covid-19 emergency, 1 died and 1 moved to another structure). *N* = 186 patients (46.6%) were receiving at least one concomitant systemic treatment for PsA (most prescribed therapies: methotrexate, 28.1%; NSAID, 12.3%; systemic corticosteroids -oral or injected-, 10.8%), and *N* = 32 patients (8.0%) were receiving topical therapies during the observation period.

Patients might receive any of the biological therapies approved and reimbursed by the National Healthcare Service, at the time of the study. At enrolment, *N* = 186 patients (46.6%) were naïve to biological treatment and *N* = 323 (81.0%) received one biologic therapy during study. The most frequently used biologic medications for PsA in the eligible population were TNFi drugs (adalimumab (originator and biosimilar): 17.8%, *N* = 71; etanercept (originator and biosimilar): 16.5%, *N* = 66; certolizumab: 9.8%, *N* = 39; golimumab: 5.0%, *N* = 20; infliximab (originator and biosimilar): 3.0%, *N* = 12; TOTAL TNFi drugs: 52.1%, *N* = 208) followed by IL17 inhibitors (secukinumab: 40.4%, *N* = 161), and finally IL12/23 inhibitors (ustekinumab: 7.5%; *N* = 30).

When the overall cohort was divided into the two subgroups (secukinumab and TNFi drugs), no statistical differences were observed between treatment groups that might have influenced the effectiveness, except for the proportion of bio-naive patients, which was higher in the TNFi group (Additional file 1).

For the economic analyses, *N* = 1 additional patient was excluded because of data incompleteness on drug utilization. As a result, *N* = 398 patients were included in the overall economic analysis.

Smaller groups were assessed in the cost-per-response analyses (Table 1) because not all patients were evaluated for DAS28 and ACR outcomes at 6 months and 12 months since observation start. The number of patients considered in the calculation of cost-per-response and cost-per-sustained response analyses are presented in Table 1.

**Table 1:** Number of patients considered in cost-per-response and cost-per-sustained response analyses according to response criteria

Response criterion	Cost-per -response analyses		Cost-per-sustained response analysis
	Patients evaluable at 6 months (N)	Patients evaluable at 12 months (N)	Patients evaluable at 6 months and 12 months (N)
DAS28	307	296	271
ACR20	194	189	NE
ACR50	194	191	NE

*ACR* College of Rheumatology response, *DAS28* Disease Activity Score 28 joints, *NE* Not evaluated

The total mean duration of the reference biologic treatment line was 18.6 (standard deviation -SD-: 6.5) months in the overall eligible population (*N* = 399), 18.8 (SD: 6.6) months in the secukinumab subgroup, 18.7 (SD 6.6) months in the TNFi drugs subgroup. Of the eligible patients, 97.7% (*N* = 390) had been on treatment with biologicals for ≥6 months. During the study, 4.8% of patients (*N* = 19) discontinued biologic treatment, and 8.3% (*N* = 33) switched from the reference biologic treatment to another biologic treatment; the remaining 86.9% (*N* = 347) of patients persisted in the reference biologic therapy (did not switch nor discontinued it).

### Resource consumption

During the observation period, all patients (*N* = 399, 100.0%) referred to outpatient specialists for PsA management, while only 1.8% of total patients (*N* = 7) went to the GP for a PsA-related issue. The mean rate of specialistic outpatient visits was 3.9 per patient/year (standard deviation (SD): 1.5).

Use of tests, procedures and instrumental examinations was common in the observed cohort. Most patients (80.2%; *N* = 320) received at least one test, procedure, or instrumental examination for PsA during the observation period. On average, patients received 11.2 analyses per year (SD: 8.1). The most common examinations (conducted in > 50% of total patients) were markers of inflammation, blood cell count, liver function test, and kidney function test.

The use of hospital resources (hospital admissions, ED accesses, duration of hospitalisation) during the observation period was extremely low, with only: i) 0.5% of total patients (*N* = 2) having at least one hospital admission; ii) 1.0% of total patients (*N* = 4) having at least one ED admission (*N* = 3 with one ED admission; *N* = 1 with two ED admissions). As result, the economic impact of hospital care for patients followed up during the observation period was negligible, compared with the cost of biologic drugs.

Table 2 provides a summary of resources used in the total cohort, in terms of: i) number of patients using the resource; ii) proportion of patients using the resource at least once during the observation period; iii) average rate of utilisation, per patient/year.

**Table 2:** Use of healthcare resources, by type

Resource	Number of patients using the resource (N)	Proportion of patients using the resource (%) (*N* = 399)	Frequency of use (Number per patient/year)
Specialistic outpatient visits	399	100.0%	3.90
GP visits	7	1.8%	NC
ED accesses	4	1.0%	NC
Hospitalizations	2	0.5%	NC
Day-hospital visits	0	0.0%	0
Tests/Procedures/Instrumental examinations	320	80.2%	11.20

*ED* Emergency Department, *GP* General Practitioner, *NC* Not Calculated

### Annualised costs

**Fig. 1:**
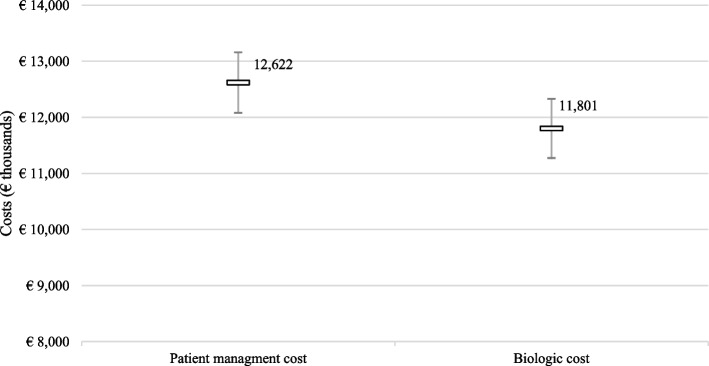
Annualised costs of patient management and biologic therapy in PsA (overall cohort; Mean, 95% C.I.). *TNFi (Tumor Necrosis Factor alpha inhibitors)*

Figure 1 shows the results of the PsA management cost analysis. On average, the annualised patient management cost in the overall cohort was €12,622. The mean overall total annualised cost was €11,725 in patients receiving secukinumab and €12,791 in the group of patients receiving TNFi.

Most of the management costs were due to the acquisition costs of biologic therapies. In fact, the percentage ratio of biologic therapies versus the total management costs ranged from 93.5% in the overall cohort to 93.0% in the TNFi group.

The cost of drug acquisition was €11,801 in the overall cohort (Fig. 1), €10,955 in the secukinumab group and €11,891 in the TNFi group. The different annual economic impact reflected the different acquisition costs.

### Cost-per-response analysis

The response rate for the DAS28 outcomes at 6 months was 71.7% in the overall cohort, 73.4% in the secukinumab subgroup) and 71.7% in the TNFi subgroup. The proportion of responders was similar at 12 months (around 70.0% in the overall cohort and both subgroups). The mean cost-per-DAS28 good/moderate response at 6 months was €13,356 in the overall cohort, while for the secukinumab and the TNFi groups was €12,661 and €13,138, respectively. At 12 months after therapy start, the mean cost-per-responder ratio was €21,912 in the overall cohort, €20,337 in the secukinumab group, and €20,874 in the TNFi group.

**Fig. 2:**
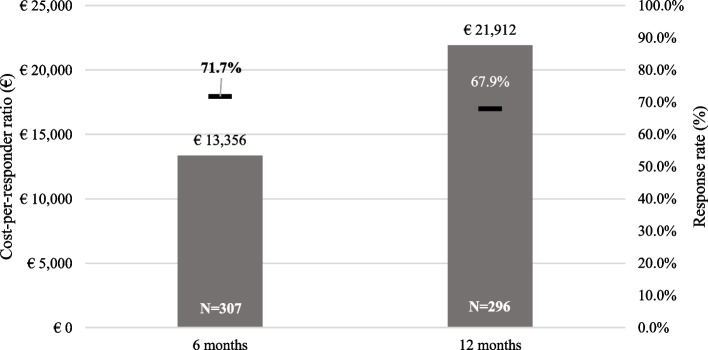
Cost per good/moderate EULAR DAS28 responder ratio at 6 and 12 months (overall cohort). *DAS28: Disease Activity Score 28 joints*

Figure 2 shows the response rate and the cost-per-responder ratios at 6 months and 12 months according to EULAR DAS28 criteria.

Complete results of the cost-per-response analysis (EULAR DAS28 and ACR criteria) in the overall population and in the secukinumab and the TNFi subgroups are showed in Table 3.

**Table 3:** Results of the cost-per-response analysis after 6 and 12 months

Outcome	Parameter	Month	Overall	Secukinumab	TNFi
DAS28 (good/moderate)	Number of evaluated patients (N)	6	307	128	159
		12	296	125	148
	Cost-per-patient (€)	6	€9571	€9298	€9420
		12	€14,879	€14,155	€14,668
	Response rate (%)	6	71.7%	73.4%	71.7%
		12	67.9%	69.6%	70.3%
	Cost-per-responder ratio (€)	6	€13,356	€12,661	€13,138
		12	€21,912	€20,337	€20,874
ACR20	Number of evaluated patients (N)	6	194	75	105
		12	189	74	100
	Cost-per-patient (€)	6	€9804	€9658	€9377
		12	€14,877	€14,674	€14,188
	Response rate (%)	6	41.2%	45.3%	39.0%
		12	34.9%	35.1%	36.0%
	Cost-per-responder ratio (€)	6	€23,775	€21,305	€24,016
		12	€42,604	€41,765	€39,412
ACR50	Number of evaluated patients (N)	6	194	75	105
		12	191	75	101
	Cost-per-patient (€)	6	€9804	€9658	€9378
		12	€14,832	€14,612	€14,158
	Response rate (%)	6	29.4%	33.3%	26.7%
		12	26.7%	29.3%	25.7%
	Cost-per-responder ratio (€)	6	€33,368	€28,975	€35,166
		12	€55,548	€49,815	€54,997

*ACR* College of Rheumatology response, *DAS28* Disease Activity Score 28 joints

Considering ACR20 and ACR50 criteria, responders were somewhat more with secukinumab at 6 months. At 12 months the ACR20 and ACR50 response rates for the overall cohort, secukinumab and the TNFi subgroups became more similar. At 6 months the cost-per-responder ratio ranged between €9804- €33,368 in the overall cohort, €9658 - €28,975 in the secukinumab group and €9377 - €35,166 in the TNFi group (Table 3) and at 12 months between €14,832 - €55,548 in the overall cohort, €14,612 - €49,815 in the secukinumab group and €14,158 - €54,997 in the TNFi group, depending on the response criteria (Table 3).

Two main factors influence the non-linear increase of cost-per-responder at 12 months compared to the 6 months cost-per-responder: i) differences in the response rate, usually lower at 12 months than 6 months; ii) considering the therapy duration (6 vs 12 months) the cost-per-patients at 6 months were in proportion higher than the cost-per-patient at 12 months.

### Cost-per-sustained response analysis

**Fig. 3:**
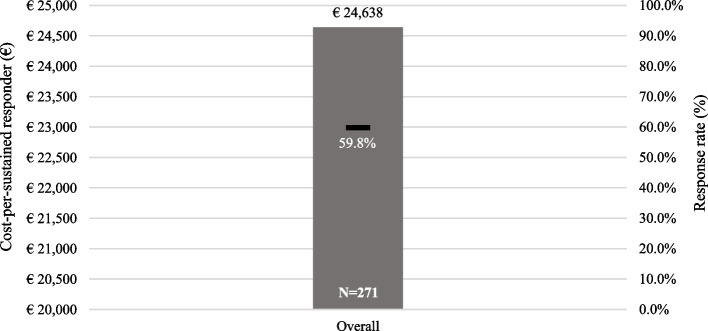
Cost per good/moderate EULAR DAS28 sustained responder ratio at 12 months (overall cohort). *DAS28: Disease Activity Score 28 joints*

Overall cost-per-sustained response are shown in Fig. 3. As expected, overall cost-per-sustained DAS28 good/moderate responder ratio at 12 months was higher than the cost-per-responder ratio for the same criteria at 12 months. Secukinumab and TNFi cost-per-sustained DAS28 responder ratio were €23,421 and €23,731, respectively, and on average €1062 lower than the overall group value (response rate 59.8% in Secukinumab and 61.7% in TNFi patients).

In the end, to better characterize the two larger treatment subgroups, we described main socio-demographic (such as age, gender, and smoking habits) and clinical characteristics (such as time since PsA diagnosis, number of prior lines received, duration of exposure to biologic treatment) at start of biologic therapy in secukinumab and TNFis subgroups to find potential confounding factors (data not shown). No difference emerged in any of the analysed variables and so the two subgroups can be considered comparable.

## Discussion

Results of the CHRONOS study show that biologic treatments were effective for the treatment of PsA in a real-world setting, with a large proportion of patients achieving good/moderate DAS28 response rates at 6 (response rate: 71.7%) and 12 months (response rate: 67.9%) after treatment start. Such findings confirmed that the effectiveness shown by biologics in pivotal RCTs is maintained in clinical practice [19, 33–36].

From an economic perspective, the study evaluated and quantified some relevant aspects of the treatment of patients with PsA: i) the economic burden and the healthcare resource utilisation; ii) the cost-per-responder ratio, in the overall cohort and in the secukinumab and TNFi subgroups; iii) the cost-per-sustained response in the overall cohort and by subgroups.

In the CHRONOS study, the annual economic impact of PsA in the overall cohort was ~€13 thousand per patient. This data is very much aligned with a recent publication in which the yearly PsA management costs per patient was approximately €14,500 - €15,500 [37]. In our study, the annualised total cost was €12,622 in the overall group of patients, €11,725 in the subgroup of patients receiving secukinumab, and €12,791 in the subgroup of patients treated with TNFi. The different annual economic impact of PsA reflected the different acquisition costs of biologic therapies. Not surprisingly, almost all healthcare direct costs were absorbed by the cost of biologics, which had a mean impact of 93.3% (range: 93.0–93.5%) on total patient management costs. Again, this cost distribution is in line with previously published articles in which the expenditure range per biologic drugs accounts for the 80–87% of the total costs [37].

The introduction of biologic therapies has determined significant benefits for patients, but at higher costs, if compared with the pre-biologic era. The distribution of costs confirmed another well-known aspect of PsA, which is in common with other autoimmune, inflammatory diseases (e.g. rheumatoid arthritis, ankylosing spondylitis, etc.): management of these conditions is almost exclusively in charge of specialised physicians, who monitor their patients on a regular basis to assess clinical response to pharmacological treatments, and prescribe typical routine tests to evaluate the course of the disease (e.g. inflammation markers, renal and liver functions, imaging). Patients rarely refer to general practitioners, and do not need of acute, hospital care, except for rare, unexpected situations (e.g. acute disease progression, treatment-related adverse events).

In the CHRONOS study, the cost-per-response ratio was chosen as cost-effectiveness indicator, instead of the more typical incremental cost-utility ratio, used in most of pharmacoeconomic evaluations [38, 39]. However, this indicator has been used previously [9, 32]. The choice was driven by several important factors: i) the objective of the CHRONOS study was to provide a picture of the economic impact of biologic treatments, and not to conduct a 1:1 pharmacoeconomic comparisons between biologics; ii) a traditional cost-utility approach was not pursued because of the CHRONOS study design, consisting of a mainly retrospective phase, during which patient utilities (required in a cost-per-QALY assessment) could not be collected; iii) a cost-utility assessment would be a valid approach in a chronic condition like PsA, when a longer time horizon (of at least 10 years) can be observed, which is not the case of the CHRONOS study.

Considering the DAS28 outcomes, as well as the ACR20 and ACR50 response criteria at 6 months, the cost-per-responder ratio was lower in the secukinumab group than the overall cohort and the TNFi group. At 12 months after therapy initiation, the cost-per-responder ratio in the secukinumab subgroup was still lower only considering the DAS28 and ACR50 outcomes. Interesting, the cost-per-responder ratio at 12 months in the overall group was higher (less favourable) than in the secukinumab as well as TNFi subgroup. Since both secukinumab and TNFi subgroups were part of the overall group, this means that the remaining treatment groups (ustekinumab) were associated with a much higher cost-per-responder ratio. Finally, in the post-hoc analysis on the cost-per-DAS28 (good/moderate) sustained responder ratio at 12 months, the observed ranked of the analysed groups was secukinumab subgroup, followed by TNFi, and the overall group. A similar response was observed in both treatment subgroups at 6 months, considering DAS28 criteria. The proportions of responders became similar for all the outcomes considered at 12 months, suggesting that in a 1-year timeframe biologics guarantee remarkable clinical benefit in most patients. However, time to response seemed depending on treatment, with earlier response rates being observed in the secukinumab subgroup. Therefore, the cost-per-sustained DAS28 response analysis showed some favourable trend for secukinumab, driven by its high response rate, which was achieved early in time (6 months). Probably, in absence of long-term data showing clear superiority of one drug versus the others, time-to-response and then cost-per-sustained response might be relevant factors influencing treatment decisions.

Moreover, findings of this economic analysis should be evaluated against some possible methodological limitations of the analysis itself. First, both annual direct healthcare costs of the disease and cost-per-response ratio might be overestimated. In fact, the cost of pharmacological treatment, which was the cost driver in this analysis, was calculated using the ex-manufacturer unit prices, extracted from the databases of the Italian Drug Agency (AIFA). Indeed, the price of these therapies might be lower in real-world, because during the procurement process manufacturers might grant discounts. Also, costs of therapies after procurement might be different by region, and even by hospital, depending on local purchase mechanisms. However, since the amount of these discounts (if any) is not clearly known (rarely in the public domain), it was preferred to use official prices to conduct the analysis. Second, only the direct costs of the disease were captured, but it is well known in literature that PsA (as well other autoimmune conditions) poses a significant economic burden on patients’ productivity [37, 40]. Despite this poses some issues of completeness of our assessment, we still believe that the analysis has great value in informing budget holders on the costs sustained by the SSN, and on consequent budget allocation. Third, the analysis is not suitable for a formal cost-effectiveness comparison among therapies. Because of the observational, non-randomized design of the study, the limited possibility of controlling for confounding factors, the complexity of the treatment pathway, the small sample size of certain subgroups (e.g. biosimilars, ustekinumab), and the uncertainty on the real acquisition costs of biologics, any attempt of comparing costs and outcomes of single therapies (to each other) might lead to misleading conclusions. For these reasons, given that most of the patients were treated with secukinumab and TNFi, we conducted subgroup analysis (on secukinumab and TNFi) without a formal comparative aim (no statistical tests were applied).

The choice of evaluating these two subgroups was driven by sample size (secukinumab group was relatively large, while it could make sense to merge TNFi drugs for their similar mechanism of action to obtain a larger group), and the relatively good balance of demographic and clinical characteristics in the two subgroups. However, while merging TNFi drugs in one single group increased sample size and made the two groups quite comparable from a clinical aspect, that unfortunately increased the heterogeneity of costs (originators and biosimilars might have different acquisition costs), thus reducing the validity of a potential comparison. This would justify the choice of not comparing therapies to each other from an economic perspective, or testing hypotheses of cost-effectiveness superiority of one therapy versus the others, but just to statistically describe costs and cost-per-response.

Nevertheless, a concomitant assessment of treatment costs against the expected therapeutic response over time, can provide physicians and payers additional insights which can complement the traditional risk-benefit profile assessment and drive treatment decisions.

## Conclusions

In conclusion, the cost-effectiveness profile of secukinumab was favourable, due to its cost (which is in line with average cost of the other biologic therapies) and the high-response rates. Cost-effectiveness results in the CHRONOS study showed that secukinumab performed better than the overall cohort (better = lower cost-per-responder ratio) in all the considered outcomes. However, this analysis cannot provide definitive conclusions on cost-effectiveness ranking of the individual therapies. Despite some potential methodological limitations of the cost-per-response analysis, we believe this approach is still an efficient, valuable, and informative economic outcome, because it allows, in principle, to detect treatment groups with the lowest cost-per-therapeutic success and rank them by economic opportunity.

## Supplementary Information

**Additional file 1.**

## Data Availability

The data that support the findings of this study are available from authors and Novartis Farma S.p.A. but restrictions apply to the availability of these data, which were used under license for the current study, and so are not publicly available. Data are however available from the authors (correspondence to Emanuela Zagni, emanuela.zagni@novartis.com) upon reasonable request and with permission of the authors and Novartis Farma S.p.A.

## Abbreviations

ACR: American College of Rheumatology
AE: Adverse Event
AIFA: Italian Drug Agency
CRP: C-Reactive Protein
DAS: Disease Activity Score
DMARDs: Disease-Modifying AntiRheumatic Drugs
ED: Emergency Department
ESR: Erythrocyte Sedimentation Rate
EULAR: European League Against Rheumatism
GH: General Health
GP: General Practitioner
NHS: National Health System
NSAIDs: NonSteroidal Anti-Inflammatory Drugs
PsA: Psoriatic Arthritis
QALY: Quality Adjusted Life Year
SD: Standard Deviation
SSN: Servizio Sanitario Nazionale (national healthcare service in Italy)
TNFi: Tumour Necrosis Factor alpha inhibitors
