# Supplementary material for: A real-world economic analysis of biologic therapies for psoriatic arthritis in Italy: results of the CHRONOS observational longitudinal study
Source: BMC Health Serv Res. 2022 Dec 16;22:1537. doi: 10.1186/s12913-022-08954-8 (PMC9757915; doi:10.1186/s12913-022-08954-8)
Supplement: Supplementary file 1 — Additional file 1. [file 12913_2022_8954_MOESM1_ESM.docx]

### Additional file 1.

**Socio-demographic and clinical characteristics at enrollment/start of biologic treatment under analysis (Overall and in Secukinumab and TNFis patients)**

|  | **Overall cohort (N=399)** | **Secukinumab (N=161)** | **TNFis (N=208)** |
| --- | --- | --- | --- |
| Age at enrolment (years), mean (SD) | 52.4 (11.6) | 53.4 (11.2) | 51.6 (11.6) |
| Males, N (%) | 172 (43.1) | 70 (43.5) | 90 (43.3) |
| Caucasian, N (%) | 398 (99.7) | 161 (100.0) | 208 (100.0) |
| Smoking status at enrolment*, N (%) | | | |
| Non-smoker | 236 (67.8) | 98 (69.0) | 121 (67.6) |
| Current smoker | 63 (18.1) | 27 (19.0) | 30 (16.8) |
| Previous smoker | 49 (14.1) | 17 (12.0) | 28 (15.6) |
| UNK | 51 | 19 | 29 |
| BMI classes at enrolment*, N (%) |  |  |  |
| Underweight (BMI < 18.5) | 9 (2.9) | 3 (2.5) | 6 (3.7) |
| Normal weight (BMI 18.5-24.9) | 111 (35.7) | 45 (37.2) | 57 (34.8) |
| Overweight (BMI 25-29.9) | 114 (36.7) | 43 (35.5) | 62 (37.8) |
| Obese (BMI ≥ 30) | 77 (24.8) | 30 (24.8) | 39 (23.8) |
| UNK | 88 | 40 | 44 |
| Duration of psoriasis at start of biologic treatment under analysis (years), median (25^th^-75^th^ percentile) | N=226 11.8 (4.7-22.9) | N=98  9.4 (3.7-22.4) | N=107  13.8 (5.7-21.7) |
| Duration of PsA at start of biologic treatment under analysis (years), median (25^th^-75^th^ percentile) | N=392 4.3 (2.1-10.0) | N=159  4.5 (2.2-9.2) | N=204  4.2 (2.1-10.5) |
| DAS28 ESR at start of biologic treatment under analysis, mean (SD) | N=279 4.0 (1.3) | N=128  4.1 (1.4) | N=136  3.9 (1.3) |
| DAS28 CRP at start of biologic treatment under analysis, mean (SD) | N=312 3.8 (1.2) | N=134  3.9 (1.1) | N=153  3.7 (1.2) |
| Total duration of biologic treatment under analysis (months), mean (SD) | 18.6 (6.5) | 18.8 (6.6) | 18.7 (6.6) |
| N of biologic therapies before biologic treatment under analysis**, N (%) |  |  |  |
| 0 (naïve patients) | 186 (46.6) | 69 (42.9) | 108 (51.9) |
| 1 | 133 (33.3) | 50 (31.1) | 69 (33.2) |
| 2 | 40 (10.0) | 19 (11.8) | 16 (7.7) |
| 3 | 27 (6.8) | 14 (8.7) | 11 (5.3) |
| >=4 | 13 (3.3) | 9 (5.6) | 4 (1.9) |
| N of biologic therapies received during study, N (%) |  |  |  |
| 1 | 323 (81.0) | 130 (80.7) | 171 (82.2) |
| 2 | 57 (14.3) | 24 (14.9) | 27 (13.0) |
| 3 | 16 (4.0) | 5 (3.1) | 9 (4.3) |
| 4 | 3 (0.8) | 2 (1.2) | 1 (0.5) |
| Comorbidities at start of biologic treatment under analysis, N (%) | 243 (60.9) | 99 (61.5) | 123 (59.4) |
| Hypertension | 127 (31.8) | 51 (31.7) | 64 (30.9) |
| Diabetes | 38 (9.5) | 15 (9.3) | 20 (9.7) |
| Hypercholesterolemia/dyslipidemia | 37 (9.3) | 13 (8.1) | 19 (9.2) |
| Thyroid diseases | 32 (8.0) | 14 (8.7) | 8 (3.9) |
| Type of PsA, N (%) |  |  |  |
| symmetric polyarthritis | 178 (44.6) | 79 (49.1) | 86 (41.3) |
| asymmetric oligoarthritis | 155 (38.8) | 57 (35.4) | 83 (39.9) |
| spondylitis | 81 (20.3) | 33 (20.5) | 43 (20.7) |
| predominant distal interphalangeal arthritis | 19 (4.8) | 6 (3.7) | 12 (5.8) |
| arthritis mutilans | 3 (0.8) | 2 (1.2) | 1 (0.5) |

Percentages and descriptives calculated over the total number of eligible patients treated with Overall Cohort (N=399) Secukinumab (N=161) or TNFis (N=208), if not otherwise specified.

*Percentages computed on available responses.

UNK: Unknown.
